# Comparison of Myeloablative Versus Reduced-Intensity Conditioning Regimens for Allogeneic Hematopoietic Stem Cell Transplantation in Acute Myeloid Leukemia: A Cohort Study

**DOI:** 10.4274/tjh.galenos.2019.2018.0220

**Published:** 2019-05-03

**Authors:** Rafiye Çiftçiler, Hakan Göker, Haluk Demiroğlu, Elifcan Aladağ, Salih Aksu, İbrahim Celalettin Haznedaroğlu, Nilgün Sayınalp, Osman Özcebe, Fatma Tekin, Yahya Büyükaşık

**Affiliations:** 1 Hacettepe University Faculty of Medicine, Department of Hematology, Ankara, Turkey

**Keywords:** Acute myeloid leukemia, Allogeneic hematopoietic stem cell transplantation, Regimen

## Abstract

**Objective:**

Allogeneic hematopoietic stem cell transplantation (HSCT) is an effective treatment modality for a variety of malignant and non-malignant hematologic disorders. Myeloablative conditioning (MAC) and reduced-intensity conditioning (RIC) regimens could have different clinical outcomes. This purpose of this study was to assess the long-term outcome of MAC versus RIC regimens in patients with acute myeloid leukemia (AML) undergoing allogeneic HSCT.

**Materials and Methods:**

We retrospectively compared long-term outcomes with MAC and RIC regimens in patients with AML who underwent allo-HSCT at our tertiary transplantation center.

**Results:**

We analyzed survival outcomes after MAC-HSCT versus RICHSCT among 107 adult patients with AML diagnosed from 2001 through 2017. Of those, 44 patients underwent a MAC regimen, whereas 63 patients received a RIC regimen. The median follow-up time was 37 months (range: 6-210) for the entire group. The 3-year overall survival (OS) for RIC and MAC patients was 67% and 60%, respectively (p>0.05). The 3-year progression-free survival (PFS) for RIC and MAC patients was 88% and 77%. In multivariate analysis, the type of conditioning regimen (RIC vs. MAC) did not influence PFS (p=0.24). Acute graft-versus-host disease (GVHD) was seen in five of the RIC patients and 9 of the MAC patients. Chronic GVHD was seen in 16 of the RIC patients and 6 of the MAC patients. There was no significant difference between the two groups in terms of acute GVHD (p=0.089), but there was a significant difference between the two groups in terms of chronic GVHD (p=0.03).

**Conclusion:**

This retrospective analysis confirmed that MAC and RIC regimens had a consistently equivalent rate of OS and PFS in AML patients who underwent allo-HSCT. The choice of MAC versus RIC conditioning regimen might be decided on the basis of patient and disease characteristics.

## Abstract (tr)

**Amaç:**

Allojenik hematopoetik kök hücre nakli (AHKHN), çeşitli malign ve malign olmayan hematolojik hastalıklar için etkili bir tedavi yöntemidir. Myeloablatif hazırlama rejimi (MHR) ve düşük yoğunlukta hazırlama rejimleri farklı klinik sonuçlara sahip olabilir. Bu çalışmanın amacı, AHKHN uygulanan akut myeloid lösemili (AML) hastalarda myeloablatif ve indirgenmiş yoğunlukta hazırlama rejimlerinin uzun dönem sonuçlarını değerlendirmektir.

**Gereç ve Yöntemler:**

Üçüncü basamak transplantasyon merkezimizde myeloablatif ve indirgenmiş yoğunlukta hazırlama rejimleri ile AHKHN uygulanan AML’li hastalarda uzun dönem sonuçları geriye dönük olarak karşılaştırıldı.

**Bulgular:**

2001-2017 yılları arasında AML tanısı almış 107 erişkin hastada AHKHN sonrasında sağkalım sonuçları analiz edildi. Ortanca takip süresi tüm hastalar için 37 aydı (6-210). İndirgenmiş yoğunlukta ve MHR alan hastalar için 3 yıllık genel sağkalım (GS), sırasıyla %67 ve %60 saptandı (p>0,05). İndirgenmiş yoğunlukta ve MHR alan hastalar için 3 yıllık progresyonsuz sağkalım (PS) %88 ve %77 olarak saptandı. Multivariate analizde hazırlama rejiminin PS’yi etkilemediği gözlendi (p=0,24). İndirgenmiş yoğunlukta hazırlama rejimi alan hastaların 5’inde ve MHR alan hastaların 9’unda akut graft-versus-host hastalığı (GVHH) gözlendi. İndirgenmiş yoğunlukta hazırlama rejimi alan hastaların 16’sında ve MHR alan hastaların 6’sında kronik GVHH gözlendi. İki grup arasında akut GVHH açısından anlamlı fark yoktu (p=0,089), ancak iki grup arasında kronik GVHH açısından anlamlı fark vardı (p=0,03).

**Sonuç:**

Bu retrospektif analizde, indirgenmiş yoğunlukta yada MHR rejimi ile AHKHN uygulanan AML hastalarında GS ve PS arasında anlamlı farklılık saptanmadı. AHKHN öncesi hazırlama rejimine hastaya ve hastalığın özelliğine göre karar verilmelidir.

## Introduction

Allogeneic hematopoietic stem cell transplantation (allo-HSCT) is a therapy with curative potential in patients with acute myeloid leukemia (AML) as well as other hematologic neoplastic disorders [1]. The therapeutic outcome of allo-HSCT lies in the balance of the risk of the myelotoxic conditioning regimen before allo-HSCT and an immunological graft-versus-leukemia effect of donor cell reactivity against host malignant cells. In AML patients, complete remission (CR) can be achieved with induction chemotherapy in almost 65% of cases. However, the 5-year progression-free survival (PFS) rate is usually lower than 50% with conventional chemotherapy [2,3,4]. Recipient age, disease status at the time of allo-HSCT, donor type, cytogenetics of the AML patients, and hematopoietic cell transplantation (HCT)-comorbidity index contribute to the outcome variables in both myeloablative conditioning (MAC) and reduced-intensity conditioning (RIC) [5]. RIC regimens could decrease toxicities related to allo-HSCT with an acceptable relapse rate. Therefore, RIC has extended the approach of allo-HSCT in AML to include patients who are not eligible candidates for standard allo-HSCT because of their advanced age and/or comorbidities [6,7,8,9]. Despite the common use of RIC allo-HSCT for the treatment of AML patients, few randomized clinical trials have compared the survival outcomes between RIC and MAC in AML patients for allo-HSCT [10,11,12]. Furthermore, several previous studies comparing the survival outcomes of RIC and MAC allo-HSCT in AML patients have reported contradictory results. Thus, we retrospectively compared long-term outcomes of AML patients who received MAC and RIC regimens for allo-HSCT at our tertiary transplantation center. Additionally, we analyzed the patient characteristics, disease and transplantation characteristics, and incidences of acute and chronic graft-versus-host disease (GVHD). This is a retrospective study. It gives insight about the almost equivalent efficacy of RIC allo-HSCT in comparison to MAC allo-HSCT.

## Materials and Methods

### Study Design, Data Collection, and Supportive Care

Our study was performed in a retrospective manner. One hundred and seven patients with AML who received allo-HSCT in our tertiary transplant center between the years of 2001 and 2017 were evaluated. RIC and MAC patients were transplanted during the same period. Patients who had intermediate or adverse cytogenetic risk scores according to the European LeukemiaNet classification [13] and who failed the first induction chemotherapy or relapsed after complete remission underwent allo-HSCT. Patients without t(8;21), inv 16, t(15;17), and t(2;5) received allo-HSCT. Patients who had a performance status between 0 and 2 by Eastern Cooperative Oncology Group (ECOG) criteria also underwent allo-HSCT [14]. Patients received antiviral prophylaxis against herpes simplex and varicella zoster, and prophylaxis against *Pneumocystis jirovecii*, for 6 months after allo-HSCT. As a result of application standards of the hospitals of our tertiary center, it was confirmed from patient records that all studied patients gave informed consent at the time of admission to the hospital and before the administration of allo-HSCT.

### Patient, Disease, and Transplant Characteristics

In this study, there were 59 males and 48 females with a median age of 45 (range: 20-66) years at the time of transplantation. Stem cells were obtained from HLA-matched related donors. Donor peripheral blood stem cells were mobilized by granulocyte colony-stimulating factor. Peripheral blood stem cells were used for all patients who underwent allo-HSCT. The indications for selecting the RIC regimen were as follows: inadequate liver, kidney, or cardiac functions (defined as serum transaminase levels >3 times the upper limit of normal reference value, total bilirubin >2 mg/dL, creatinine clearance <60 mL/min, left ventricular ejection fraction <50%); serious fungal infection before allo-HSCT; ECOG performance status of >2; and the patient’s refusal of the MAC regimen before allo-HSCT.

### Conditioning Regimens

Mainly the busulfan, fludarabine, and antithymocyte globulin (BU/FLU/ATG) RIC regimen was preferred. An intravenous BU/FLU/ATG regimen was applied for 63 patients, consisting of intravenous (i.v.) fludarabine at 50 mg/kg over 30 min for 6 consecutive days, 9 mg/kg or less oral busulfan (or intravenous equivalent q6h for 2 consecutive days), and ATG at 5 mg/kg/day for 3±1 consecutive days [15]. Phenytoin was given to prevent busulfan-induced seizures. The preferred MAC regimen was i.v. cyclophosphamide at 60 mg/kg daily for 2 days and busulfan at >8 mg/kg orally (or intravenous equivalent more than 0.8 mg/kg i.v. infusion q6h) for 4 days [16]. The other myeloablative conditioning regimen was i.v. BU at >0.8 mg/kg q6h for 4 days, plus i.v. FLU with ATG.

### GVHD Prophylaxis and Grading

All patients received standard cyclosporine A (CsA) and methotrexate therapy for GVHD prophylaxis. Usually, tapering of immune suppression was initiated at 3 months after allo-HSCT in the absence of acute or chronic GVHD, with the aim of stopping it by approximately 6 months after HSCT. Acute and chronic GVHD were graded according to the related consensus criteria [17,18].

### Statistical Analysis

SPSS 25 (IBM Corp., Armonk, NY, USA) was used to perform statistical analyses. The variables were investigated using visual (histograms, probability plots) and analytical methods (Kolmogorov-Smirnov/Shapiro-Wilk test) to determine whether they were normally distributed or not. Statistical comparisons were made using chi-square tests for categorical data. The Student t-test (for two independent samples) was used for comparison of continuous numerical data. Survival analyses were made using the Kaplan-Meier test. Multivariate analyses of predictors of survival were performed using the Cox regression test. Parameters with p≤0.20 in univariate tests were included in the multivariate analysis, and p<0.05 was considered to indicate statistical significance. Cumulative incidences of relapse and non-relapse mortality were calculated by means of the statistical software environment R version 2.15.2 [19].

### Ethics

All of the ethical considerations were strictly followed in accordance with the 1964 Helsinki Declaration in the Hacettepe University Faculty of Medicine. As standard care/action of the hospitals of the Hacettepe University Faculty of Medicine, it has been recognized from the patient records that all of the studied patients gave informed consent at the time of admission to the hospital and before the administration of chemotherapy and other relevant diagnostic/therapeutic standards of care.

## Results

### Patient and Allo-HSCT Characteristics

Patient and transplant characteristics for all patients with AML are summarized in Table 1. One hundred and seven patients with AML underwent allo-HSCT using peripheral blood stem cells from matched related donors. The RIC regimen was applied to 63 patients (58.8%) and the MAC regimen was applied to 44 patients (41.2%). The median age at transplantation was 51 (23-66) years for RIC patients and 43 (20-63) years for MAC patients. The median age at transplantation was significantly higher in RIC patients compared to MAC patients (p=0.002). There was no statistically significant difference between the two groups for sex of the patients (p=0.28). There was also no statistically significant difference between the two groups for sex combinations of patients and donors (p=0.84). The number of patients graded with ECOG performance status 0, 1, and 2 was 1 (1.6%), 39 (61.9%), and 23 (36.5%) for RIC patients, while 3 (6.8%) and 41 (93.2%) MAC patients were graded with performance status 0 and 1, respectively [14]. There was a statistically significant difference between the two groups for ECOG performance status (p<0.001). Patients who received the MAC regimen had better ECOG performance status than patients who received the RIC regimen. Cytogenetic analyses were present for 96 patients: 42 (66.7%) patients were classified in the intermediate-risk group and 14 (22.2%) patients were in the adverse-risk group among RIC patients, while 35 (79.5%) patients were classified in the intermediate-risk group and 5 (11.4%) patients were in the adverse-risk group for MAC patients according to the European LeukemiaNet classification [13]. There was no statistically significant difference between the two groups for karyotype analyses (p=0.25). The CD34+ cell counts were 7.5 (±4.0)x10^6^/kg for RIC patients and 9.3 (±5.6)x10^6^/kg for MAC patients (p=0.06). Cytomegalovirus seropositivity statuses of the patients and donors were similar between the two groups receiving RIC and MAC regimens (p=0.52).

The HCT-comorbidity index of patients was statistically significantly different between the two groups (p<0.001). There was no statistically significant difference in terms of the disease risk index between the two groups of patients (p=0.31). The disease statuses of RIC and MAC patients during transplantation were similar. Primary graft failure was not observed in those patients. A total of 22 (34.9%) RIC recipients and 15 (34.1%) MAC recipients died during the follow-up period (p=0.93). The clinical characteristics of RIC and MAC patients are summarized in Table 2.

### Survival Outcomes

**Figure 1:**
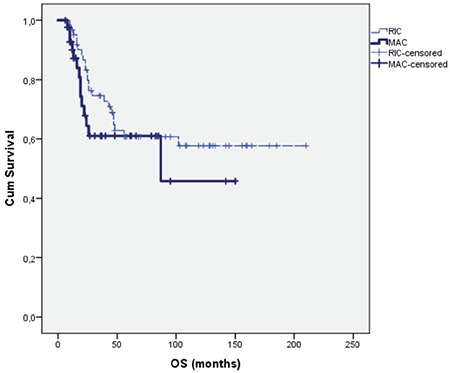
The overall survival for RIC and MAC patients (p=0.29). OS: Overall survival, RIC: reduced-intensity conditioning, MAC: myeloablative conditioning.

The median follow-up period was 37 months (range: 6-210) for the all patients. The 6-month overall survival (OS) was 93% in RIC patients compared to MAC patients with 82%, with no statistically signiﬁcant difference. The 3-year OS for RIC and MAC patients was 67% and 60%, respectively (p=0.22). The 5-year OS rates for RIC and MAC patients were both 60%. The OS for RIC patients was 135±12 versus 88±13.0 months for MAC patients with no statistically signiﬁcant difference, as shown in Figure 1 (p=0.29).

**Figure 2:**
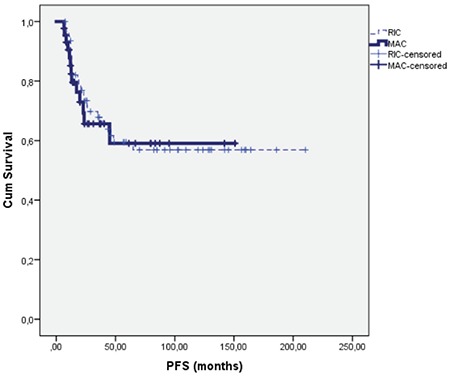
The progression-free survival for RIC and MAC patients (p=0.78). PFS: Progression-free survival, RIC: reduced-intensity conditioning, MAC: myeloablative conditioning.

The 3-year PFS for RIC and MAC patients was 88% and 77%. The type of conditioning regimen did not influence 3-year PFS (p=0.24). The 5-year PFS for RIC and MAC patients was 59% and 59%, respectively. The PFS for RIC patients was different from the PFS for MAC patients (130±12 versus 96±12 months), but no statistically signiﬁcant difference was observed, as shown in Figure 2 (p=0.78).

### Non-relapse Mortality

**Figure 3:**
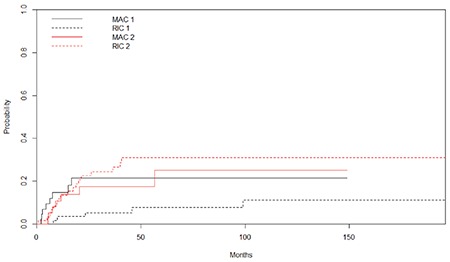
Cumulative incidence of non-relapse mortality for reduced-intensity conditioning (RIC) and myeloablative conditioning (MAC) regimens (MAC 1 and RIC 1) (p=0.027) and cumulative incidence of relapse for RIC and MAC regimens (MAC 2 and RIC 2) (p=0.496). RIC: Reduced-intensity conditioning, MAC: myeloablative conditioning.

Non-relapse mortality (NRM) was more frequent in the MAC patients than RIC patients (7/44, 15.9% vs. 5/63, 7.9%, p=0.027). The major causes of NRM were infections (5 vs. 5), GVHD (1 vs. 0), and heart attack (1 vs. 0) in the MAC and RIC patients, respectively. The cumulative relapse incidence was not statistically significantly different between RIC and MAC patients (p=0.49) (Figure 3).

### Acute and Chronic GVHD

**Figure 4:**
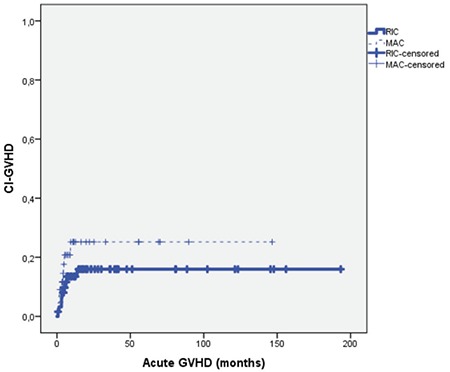
Cumulative incidence plot of acute graft-versus-host disease for reduced-intensity conditioning and myeloablative conditioning regimens (p=0.22). GVHD: Graft-versus-host disease, CI: cumulative incidence, RIC: reduced-intensity conditioning, MAC: myeloablative conditioning.

**Figure 5:**
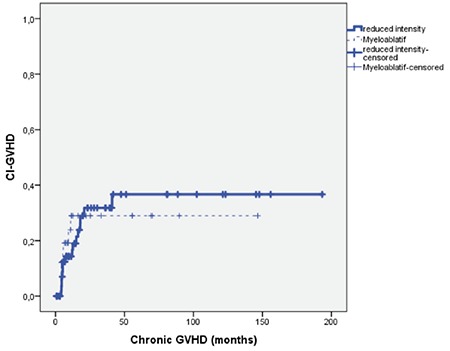
Cumulative incidence plot of chronic graft-versus-host disease for reduced-intensity conditioning and myeloablative conditioning regimens (p=0.79). GVHD: Graft-versus-host disease, CI: cumulative incidence.

Nine of the RIC patients (14.3%) and 9 of the MAC patients (20.5%) developed acute GVHD. Sixteen of the RIC patients (25.4%) and 8 of the MAC patients (18.2%) developed chronic GVHD. There was no statistically significant difference between the RIC and MAC patients in terms of acute (p=0.40) and chronic GVHD (p=0.37). The cumulative incidence rates of acute (p=0.22) and chronic GVHD (p=0.79) in the RIC and MAC groups did not differ to a statistically significant extent (Figures 4 and 5; Table 3).

### Cox Regression Analysis

In univariate analyses the parameters that affected OS were development of chronic GVHD (p=0.04) and CD34+ counts (p<0.001), as shown in Table 4. Cox regression analysis revealed CD34+ cell counts and development of chronic GVHD as parameters to predict OS.

In univariate analyses the parameters that affected PFS were cytogenetics of the patients (p=0.08), disease risk index (p=0.006), and HCT-comorbidity index of the patients (p=0.10), as shown in Table 4. However, Cox regression analysis revealed no parameters to predict PFS.

## Discussion

Allo-HSCT is an effective treatment modality for several malignant and non-malignant hematologic disorders [1]. The intensity of conditioning regimens can vary substantially. When selecting the right conditioning regimen for AML patients, disease-related factors such as diagnosis and remission status and patient-related factors including age, donor availability, and presence of comorbid conditions are the parameters to be considered [20]. 

Recently a randomized study was performed in patients with AML or MDS in remission comparing RIC conditioning to MAC conditioning and using related or unrelated donor grafts. The trial closed with 272 enrolled patients due to excess relapse in the RIC arm. RIC was associated with more relapse, lower NRM, lower relapse-free survival, and, in the AML subgroup, lower OS. The conclusion of the study was that MAC conditioning should be the standard of care for fit patients with AML or MDS [12].

Choosing the proper conditioning remains challenging, given the need to balance the risk of relapse with the risk of transplantation-related mortality. The main finding of our study was that RIC and MAC yielded similar outcomes (OS and PFS), even though patients in the RIC arm were somewhat older. 

The European Group for Blood and Marrow Transplantation analyzed survival outcomes of patients with AML older than 50 years treated with HLA-matched sibling allo-HSCT after RIC or MAC regimens. Despite the older age of the RIC patients, grade 2-4 acute GVHD and transplant-related mortality were significantly lower after the RIC regimen. However, there was no statistically significant difference in OS and PFS for patients receiving either the MAC or the RIC regimen, regardless of the status of the disease at the time of transplantation [21]. Goker et al. [22] compared survival outcomes of patients transplanted with RIC versus MAC regimens. They showed that the MAC regimen was associated with lower OS and PFS. Additionally, they showed an apparent favorable effect of the RIC regimen as a lower acute GVHD rate. On the other hand, it had a higher rate of chronic GVHD.

GVHD and relapses remain major causes of mortality during HSCT [23,24]. The RIC regimen caused less tissue damage and lower levels of inﬂammatory cytokines, which may explain the lower incidence of severe GVHD following RIC conditioning [23,25,26,27]. It has been demonstrated that the incidence of acute GVHD was related to the intensity of the conditioning regimen. RIC, which consists of total body irradiation (2 Gy) with or without fludarabine, was reported to reduce the incidence of severe acute GVHD compared with MAC [27]. Cutler et al. [28] showed that allogeneic peripheral blood stem cell transplantation was associated with a greater degree of acute and chronic GVHD than bone marrow transplantation and this may be related to lower rates of relapse. Peripheral blood grafts were used for all allo-HSCT procedures in our patients. This study showed that the incidence of acute and chronic GVHD was similar between RIC and MAC regimen groups. Reported incidence rates range from 9% to 50% in patients who receive allo-HSCT from a genotypically HLA-identical sibling [29,30]. We followed our patients closely and made sure that they used their immunosuppressive drugs regularly. Therefore, this study may show a lower rate of GVHD. Future studies will reveal the cause of chronic GVHD seen to be lower in MAC regimens than in RIC regimens in this study.

## Conclusion

In this retrospective evaluation, RIC allo-HSCT outcomes were similar when compared to the MAC allo-HSCT outcomes in our patients with AML. MAC and RIC regimens were similar in terms of OS and PFS in AML patients with allo-HSCT. The incidence of acute and chronic GVHD was also similar between the two groups. NRM was more frequent in the MAC patients than RIC patients. Relapse rate was similar between the RIC and MAC patients. This study has some limitations. First of all, the study was retrospective. There were imbalances between the patient populations, which is known as a weakness of this type of retrospective study. Local standards also changed from 2001 to 2017. Therefore, spanning a long time period was a limitation of this study. In conclusion, the conditioning regimen should be tailored and chosen based on the disease and individual patient characteristics. Future powerful randomized clinical trials could further elucidate the type of conditioning to be used and tailored on a per patient basis.

## Figures and Tables

**Table 1:**
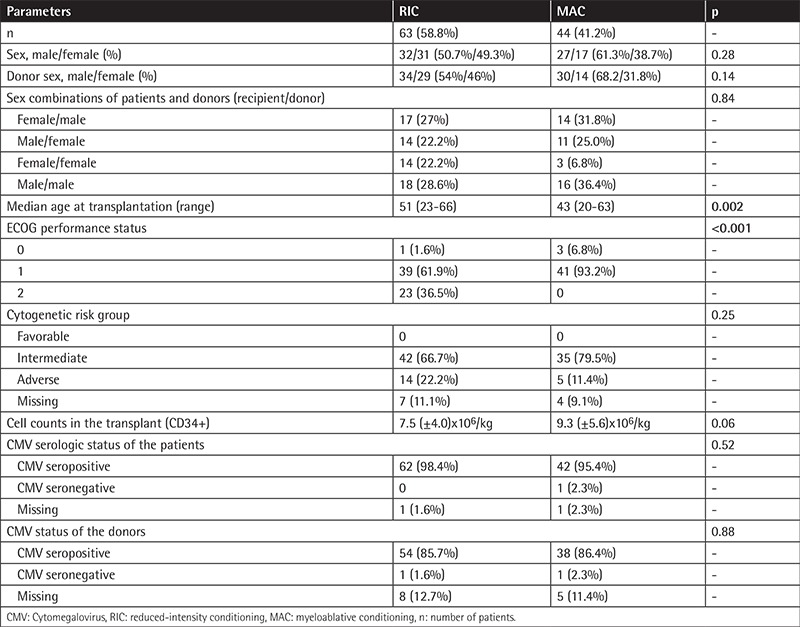
Patient and transplantation characteristics of reduced-intensity conditioning and myeloablative conditioning patients.

**Table 2:**
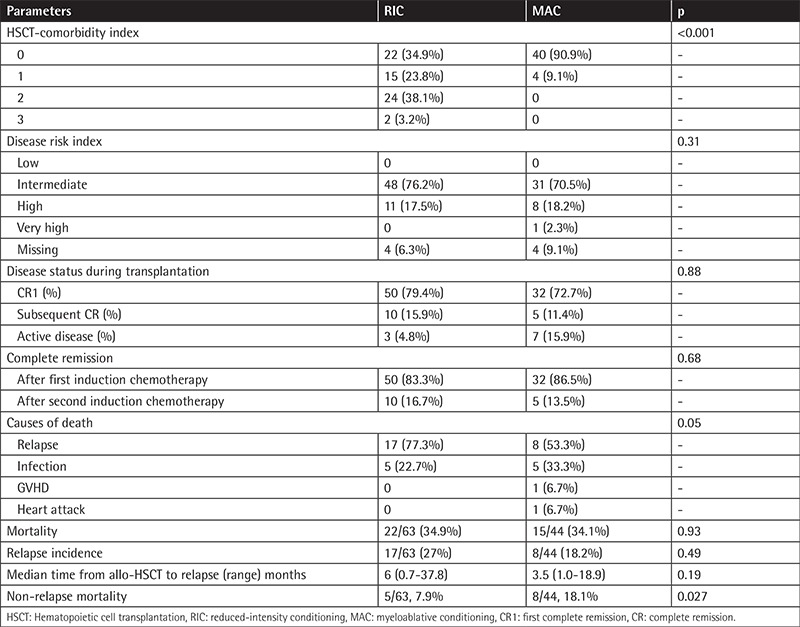
Disease characteristics of the reduced-intensity conditioning and myeloablative conditioning patients.

**Table 3:**
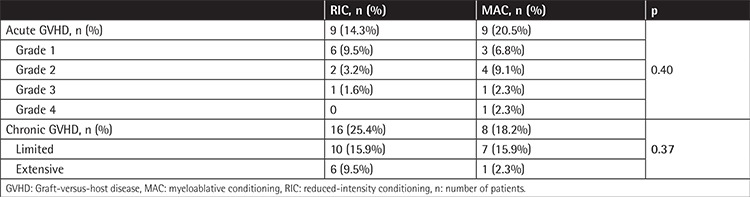
Acute and chronic Graft-versus-host disease in patients treated with myeloablative conditioning and reduced-intensity conditioning regimens.

**Table 4:**
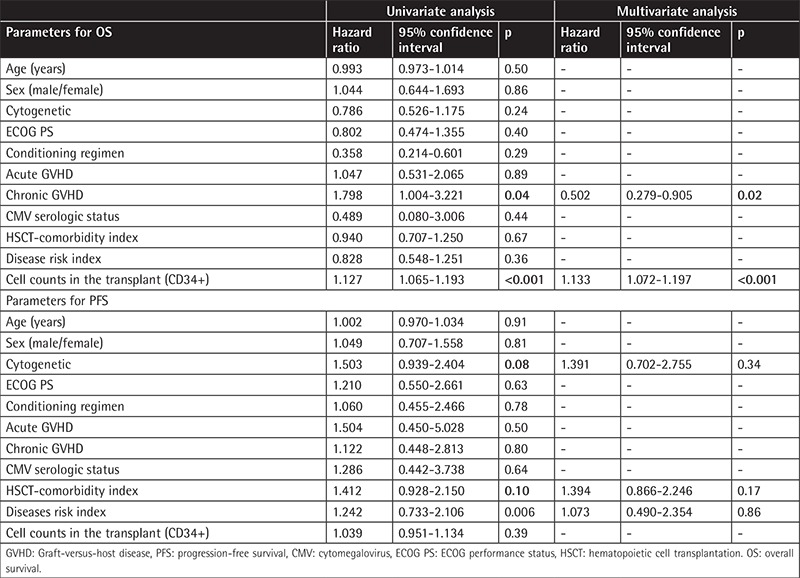
Univariate and multivariate analysis of overall survival and progression-free survival (univariate comparisons with p<0.20 were included in multivariate analysis in which statistical significance threshold was acknowledged as p<0.05).
